# Real-World Experiences With Facilitated Subcutaneous Immunoglobulin Substitution in Patients With Hypogammaglobulinemia, Using a Three-Step Ramp-Up Schedule

**DOI:** 10.3389/fimmu.2021.670547

**Published:** 2021-04-27

**Authors:** Nina B. Hustad, Hanna M. Degerud, Ingrid Hjelmerud, Mai S. A. Fraz, Ingvild Nordøy, Marius Trøseid, Børre Fevang, Pål Aukrust, Silje F. Jørgensen

**Affiliations:** ^1^ Medical Day-Unit, Division of Surgery, Inflammatory Diseases and Transplantation, Oslo University Hospital, Rikshospitalet, Oslo, Norway; ^2^ Section of Clinical Immunology and Infectious Diseases, Department of Rheumatology, Dermatology and Infectious Diseases, Oslo University Hospital, Rikshospitalet, Oslo, Norway; ^3^ Research Institute of Internal Medicine, Division of Surgery, Inflammatory Diseases and Transplantation, Oslo University Hospital, Rikshospitalet, Oslo, Norway; ^4^ Institute of Clinical Medicine, University of Oslo, Oslo, Norway

**Keywords:** SCIG, PID, CVID, fSCIg, hypogammaglobulinemia, immunoglobulin therapy, IVIG, home infusions

## Abstract

Immunoglobulin replacement therapy with facilitated subcutaneous immunoglobulin (fSCIg) can be self-administrated at home and given at longer intervals compared to subcutaneous immunoglobulin (SCIg) therapy, but real-word experience of home-based fSCIg therapy is limited. Herein we present our real-word clinical experiences with home-based fSCIg therapy using a three-step ramp-up schedule. We registered data from all patients with immunodeficiency starting fSCIg from 01.01.2017 to 31.12.2019. For comparison we also included patients starting conventional SCIg training. Fifty-four patients followed for a median of 18 months (IQR 12, range 0–40), received fSCIg training, and 84 patients received conventional SCIg training. Out of 54 patients starting with fSCIg, 41 patients had previous experience with conventional SCIg therapy, and the main reason for starting fSCIg was ‘longer intervals between therapies’ (n=48). We found an increase in training requirement for fSCIg (3 ± 1 [2-9] days) compared to conventional SCIg (2 ± 0 [1-7] days), *P*< 0.001 (median ± IQR, [range]). For fSCIg training, IgG levels were stable from baseline (8.9 ± 2.3 g/L), 3-6 months (10.2 ± 2.2 g/L) and 9-12 months (9.9 ± 2.3 g/L), *P*= 0.11 (mean ± SD). The most common side-effect was: ‘rubor around injection site’ (n=48, 89%). No patients experienced severe adverse events (grade 3-4). Thirteen patients (24%) discontinued fSCIg therapy due to local adverse events (n=9), cognitive/psychological difficulties (n=6) and/or systemic adverse events (n=3). In conclusion, fSCIg training using a three-step ramp-up schedule is safe and well tolerated by the majority of patients, but requires longer training time compared to conventional SCIg.

## Introduction

Immunoglobulin replacement therapy (IgRT) has been the backbone of treatment in patients with primary and secondary hypogammaglobulinemia for decades. The main indication for IgRT in these patients is to prevent infections with encapsulated bacteria (i.e., *Streptococcus pneumonia, Moraxella catarrhalis and Haemophilus influenza)* in the respiratory tract that could lead to irreversibly lung damage. IgRT has been shown to reduce the severity and number of infections (1), and probably enhances survival in these patients (2).

IgRT is given either subcutaneous (s.c.), using a battery powered pump, or intravenously (IV). The main advantages of conventional subcutaneous immunoglobulin (SCIg) compared to intravenous Immunoglobulin (IVIg) are less severe systemic complications, more stable IgG levels (avoiding “wear off” effect as seen with IVIg) and that it can be self-administrated at home, given appropriate training (3, 4). The major disadvantages of conventional SCIg compared to IVIg are the frequency of therapy and local adverse reactions (5).

In 2014, facilitated subcutaneous immunoglobulin (fSCIg) with the commercial name HyQvia (Takeda Pharmaceutical Company Limited US Inc., Tokyo, Japan) was launched for patients with primary immunodeficiency (PID). It uses a new method for administrating SCIg therapy. Previously, the volume injected by conventional s.c. therapy was limited by the inherent resistance to bulk fluid flow through the extra cellular matrix of the s.c. tissue. The main component of the s.c. extra cellular matrix responsible for this resistance is hyaluronan. HyQvia consists of IgG 10% in combination with recombinant human hyaluronidase (rHuPH20). Hyaluronidase has the ability to counteract hyaluronic acid, and facilitates the permeability of bulk fluid flow through the s.c. tissue. When rHuPH20, a soluble from of naturally occurring human hyaluronidase, is injected into the tissue it depolymerizes/cleaves hyaluronan and increases the permeability of the extra cellular matrix trough nanometer-sized micro channels (6, 7). This mechanism increases the dispersion and absorption of IgG, and is the reason why fSCIg can be given at large volumes and thereby longer intervals compared to conventional SCIg (7). fSCIg therapy merges some of the major advantages from both IVIg and SCIg as it is given at 3-4 weeks interval as a s.c. injection, and it can be administrated at home by the patients themselves. The safety and efficiency of HyQvia have been validated in clinical trials for patients with PID (6, 8, 9), but the literature is almost devoid of real-world experience with fSCIg therapy for home-use. One of the main differences between conventional SCIg and fSCIg is that the volume given with each infusion with HyQvia is considerably larger (x10) than with conventional SCIg. The potential increase in side effects, due to the increased volume injected, was a concern before initiating treatment with fSCIg at our department and was evaluated in the present study.

A 7-week ramp-up period suggested by the manufacturer of HyQvia (10) is unpractical in real-word use. Wasserman et al. recently reported that 37 out of 38 PID patients from seven US Immunological Clinics received HyQvia differing from prescribing guidelines (11). In the present survey we used a modification of the 7-week ramp-up period that has been recommended by the manufacturer, and altered it to a three-step ramp-up period.

Here, we present our real-word clinical experiences with home-based self-administrated fSCIg therapy in 54 patients with primary and secondary hypogammaglobulinemia from 2017-2019, using a three-step ramp-up schedule. By comparison, we also evaluated 84 patients that received conventional SCIg training in the same time period.

## Materials and Methods

### Survey Design

We designed a survey to monitor training and outcome for home-based SCIg therapy (fSCIg as compared with conventional SCIg). All patients with primary or secondary immunodeficiency that started training for home-based SCIg therapy at the Medical Day-unit (a part of Section of Clinical Immunology and Infectious Diseases) at Oslo University Hospital, between 2017 and 2019, were registered. The registration period for adverse events (AEs) (12) and termination of therapy was until 26.05.2020, ensuring that the patients starting SCIg at the end of 2019 were followed up for at least 22 weeks. The aim of the survey was to aid future decision-making regarding home-based s.c. therapy. Ethical approval was deemed unnecessary by the Institution’s Research and Development department at Oslo University Hospital. There was no external funding for this survey, in particular no funding from Takada, the manufacturer of the fSCIg.

### Patients Offered HyQvia

Most patients were informed about the option of starting/switching to fSCIg by doctors and nurses. In addition, a few patients asked themselves to switch to, or start, fSCIg. Not all patients were systematically offered the option of HyQvia due to individual variation between doctors/nurses to discuss this option. ‘Pros and cons’ for fSCIg were discussed in relation to other IgRTs. In the end, it was the patients’ own decision to start with fSCIg or not.

#### Local Adjustments to Dosing Guidelines Using a Three-Step Ramp-Up Schedule

**Figure 1:**
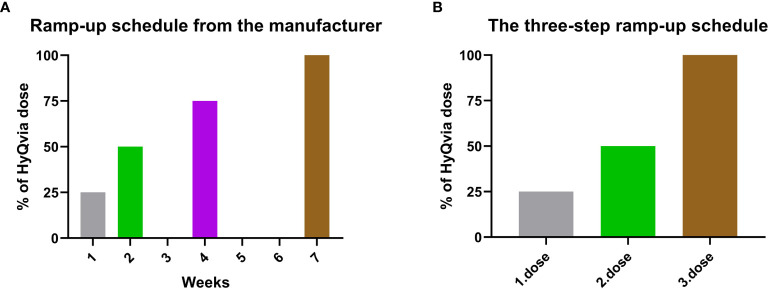
The American HyQvia dosing guidelines compared to the three-step ramp-up schedule. **(A)** The American HyQvia dosing guidelines showing that the 7-week ramp-up period is planned at set weeks. **(B)** The three-step ramp-up schedule is not given at set weeks. Dose 1 and 2 can be given with a minimum of one day apart, and the third dose is given after the patients have received their electrical infusion pumps from the local ‘Medical equipment supplier’, which often takes a few weeks. The 75% of total HyQvia dose is skipped in the three-step ramp-up schedule compared to the American HyQvia dosing guidelines.

The American HyQvia dosing guidelines include a 7-week ramp-up period (**Figure 1A**) that is unpractical in real-word use, as discussed in a recent paper by Wasserman et al. (11). Our hospital offers on-site s.c. training for patients from all parts of Norway, and therefore the training scheduled for fSCIg had to be convenient also for patients living far away from the hospital. The on-site training was aimed at home-based self-administration without health professionals present. We therefore developed an adapted version of The American HyQvia dosing guidelines (10) (**Supplementary Methods**). The HyQvia dose was given as a three-step ramp-up schedule with 25%, 50%, a 100%, of the total HyQvia dose at each step (**Figure 1B**). The major differences between the original guidelines and the three-step ramp-up schedule are that the ramp-up is not given at set weeks 1-7, making the organization of the training more practical, and that the 75% of total HyQvia dose between the dose of 50% and 100% is skipped (**Figure 1**). For details regarding s.c. training and administration, see **Supplementary Methods** and **Supplementary Tables S1a, b**.

### Registration Sheet

Three specialist-trained nurses in fSCIg therapy filled out a registration sheet for each visit from the first training session to the end of the registration period, or to the discontinuation of fSCIg.

### Data Analyses

Univariate analyses were performed using parametric (t-tests) or non-parametric methods (Mann–Whitney’s U-tests) as appropriate for continuous variables, and Fisher’s exact test for categorical variables. We used repeated measures ANOVA (Unianova) analysis for IgG levels at the three different time points. P-values were two-sided and considered significant when <0.05. Statistical analyses were performed with SPSS (IBM, Armonk, NY). Figures were made using Prism software.

## Results

### Self-Administration Training at the Medical Day-Unit 2017-2019

At the Section of Clinical Immunology and Infectious Diseases, Oslo University Hospital, Rikshospitalet, Norway we care for patients, from across the country, with primary and secondary antibody deficiency. From 01.01.2017 to 31.12.2019, 54 patients received fSCIg training with a follow-up period of 80 patient years. Eighty-four patients received conventional SCIg training in the same period. There was some overlap as eight patients received both conventional SCIg and fSCIg training, and one patient had two rounds of conventional SCIg training (one with a 20 ml pump and one with 50 ml pump). In total, 129 patients received s.c. training.

### Patients’ Characteristics

Patients’ characteristics, including diagnosis and prior Ig therapy, for patients that started fSCIg and conventional SCIg 2017-2019 are shown in **Table 1**. The patients receiving training for fSCIg were younger and a higher percentage was diagnosed with Common variable immunodeficiency compared to the patients undergoing conventional SCIg training. Most patients (41 of 54 patients, 76%) starting with fSCIg had previous experience with conventional SCIg therapy. There were significantly more patients that were treatment naïve or on IVIg therapy starting SCIg training compared to those starting fSCIg training (**Table 1**).

**Table 1:** Characteristics for patients that underwent fSCIg or SCIg training from 2017-2019.

	fSCIg, (n=54)	SCIg, (n=84)	P-value^e^
**Age ± IQR (min-max)**	37 ± 18 (20-66)	48 ± 29 (18-75)	0.019
**Male, n (%)**	18 (33)	21 (25)	0.335
**Diagnosis, n (%)**			
*CVID*	31 (57)	25 (30)	0.001
*Primary hypogammaglobulinemia^a^*	6 (11)	21 (25)	0.050
*sIgG2d*	6 (11)	12 (14)	0.796
*Bruton’s agammaglobulinemia*	1 (2)	0 (0)	–
*Secondary hypogammaglobulinemia*	6 (11)	22 (26)	0.050
*Secondary sIgG2d*	3 (6)	3 (4)	–
*Other^b^*	1 (2)	1 (1)	–
**Prior IgRT**			
*Naïve*	6 (11)	30 (36)	0.001
*SCIg*	37 (69)	–	–
*IVIg*	7 (13)	28 (33)	0.009
*SCIg & IVIg*	4 (7)	–	–
*fSCIg*	–	1 (0)	–
*I.m. Ig^c^*	–	1 (0)	–
*SCIg Pump switch^d^*	–	24 (29)	–

CVID, Common variable immunodeficiency; fSCIg, facilitated subcutaneous Immunoglobulin; SCIg, subcutaneous Immunoglobulin; IgRT, Immunoglobulin replacement therapy; IVIg, intravenous immunoglobulin; sIgG2d, selective Immunoglobulin G subclass 2 deficiency; IQR, Interquartile range; I.m., intramuscular;

^a^Patients with hypogammaglobulinemia that do not fulfil the CVID diagnosis, often because only IgG is low (IgA and IgM are normal) and with no other apparent reason for their hypogammaglobulinemia. ^b^Other; fSCIg: one patient with reduced IgG3 and IgM and frequent respiratory tract infections, SCIg: one patient with hypogammaglobulinemia secondary to Ataxia Telangiectasia.^c^We do not use i.m. Ig as IgRT. However, this patient was referred from a private practitioner who had initiated the therapy. ^d^From 20ml to 50 ml ^e^P-value is calculated if > 5 individuals in each group.

### Reason for Starting fSCIg

The total number of patients on IgRT at the Section of Clinical Immunology and Infectious Diseases from 2017-2019 was 404. Of these, 12 percent of patients transferred from other IgRT to fSCIg in the observation period.

The reasons for switching/starting fSCIg were “longer intervals between therapy” (n=48), “problems with the SCIg therapy” (n=5), “poor compliance with SCIg” (n=3), “fewer injections” (n=2), “fear of injections/needle phobia” (n=5), “problems with IV access” (n=2), “wish to try something new” (n=2), “want home treatment” (n=1) and “s.c. lumps” (n=1). For one patient the reason for starting/switching was not given.

### Training Duration Using a Three-Step Ramp-Up Schedule

**Figure 2:**
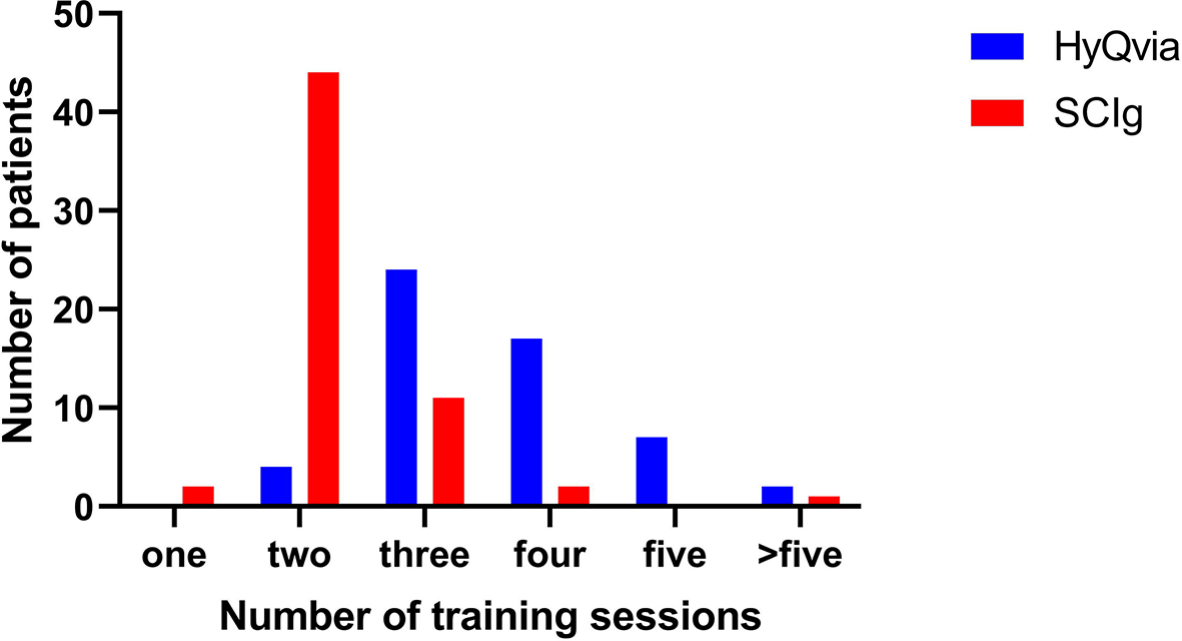
Number of teaching sessions per person for self-administration for conventional SCIg and fSCIg. There was one training session at each visit, and each visit was one day at the Medical Day-unit.

The fSCIg training was performed by three specialist-trained nurses (NBH, HMD and IH). They were all experienced in teaching conventional SCIg. The first two doses (25% and 50% of total dose, respectively) of HyQvia were given with minimum one day apart (median 7 days [range 1-51]). The third dose was scheduled after each patient had received the infusion pump. The third dose (100% of total dose) was given on average 29 (median) days after the first visits (range 12-135 days). The reason for the large variation between visits was often practical as some of the patients lived far from the hospital or that the delivery of the infusion pump was delayed. The total number of visits necessary for both fSCIg and conventional SCIg training is given in **Figure 2**. There was one training session at each visit, and each visit was one day at the Medical Day-unit. There were significantly more training visits for fSCIg (3 ± 1 [2-9] days, n=54) compared to conventional SCIg (2 ± 0 [1-7] days, n=60), *P*< 0.001, median ± interquartile range (IQR) and range (**Figure 2**). The 24 patients switching from conventional SCIg 20 ml to SCIg 50 ml pump were not included in these analyses, since this training was with a similar pump and the patients were already familiar with the procedure.

### The Dose, Interval and Injection Site of fSCIg

**Figure 3:**
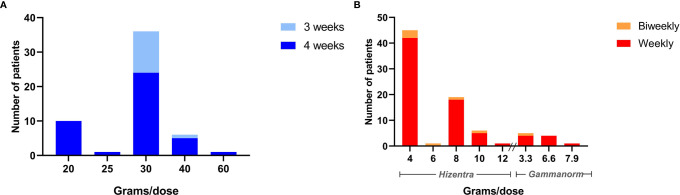
The dosage in grams of Immunoglobulin for fSCIg and conventional SCIg therapy per patient. **(A)** The number of patients on the different fSCIg dose, in grams, after the end of the training sessions with corresponding intervals in therapy. **(B)** The number of patients on each dose of conventional SCIg, Hizentra (200 mg/ml) and Gammanorm (165mg/ml), after the end of training sessions with corresponding intervals in therapy.

The dose and interval of HyQvia was calculated by converting previous weekly dose into a monthly dose and then doing some practical adjustments. For example, 20 ml Hizentra per week (equals 16 g Ig a month) and 40 ml Hizentra per week (equals 32 g Ig a month) were rounded up to 20 g (200 ml HyQvia) a month, and down to 30 g (300 ml HyQvia) a month, respectively (**Supplementary Table S2**). For the treatment-naïve patients the dose was based on weight (approximately 0.4 g per kg every 4 weeks). Sometimes the interval of therapy was reduced to three weeks to reduce volume needed with each injection or to avoid switching Ig bottles. For further details regarding dosing adjustments see **Supplementary Information** and **Supplementary Table S2**. The full doses of fSCIg and SCIg given after the training was finished are given in **Figures 3A, B**.

The total dose of IgG given per month were significantly higher in fSCIg patients (median 30 g ± 10 compared to conventional SCIg, median 16 ± 16 [median ± IQR]), *P*< 0.0001, **Supplementary Figure S1**, reflecting the higher disease burden in the patients starting fSCIg compared to SCIg.

Fifty patients injected fSCIg into the abdomen in one single injection and four patients used two injection sites (three patients injected both injections into the abdomen at different sites, and one patient used one injection in the thigh and one in the abdomen). All patients used the electric pump BodyGuard 323 (Caesarea Medical Electronics Ltd., Caesarea, Israel).

### IgG Stability When Switching IgRT Therapies

We recorded IgG levels at baseline, at 3-6 months and at 9-12 months after initiating fSCIg. Fifty-two patients had baseline IgG levels taken before initiating fSCIg treatment, mean IgG 8.34 ± 2.65 (mean ± SD, range 2.50-13.80) g/L. Since not all patients continued with fSCIg, and some patients did not come to the follow-up appointments at the assigned time, the data in terms of IgG levels is not complete. Also, the patients starting fSCIg in 2019 had not all reached the time for their follow up appointment. The treatment-naïve patients that had reduced IgG levels (< 6.5 g/L) at baseline were excluded from the analysis (n=3). In the end, thirty patients had baseline IgG levels and a corresponding IgG level at 3-6 months and/or 9-12 months. When including all three time points in the analysis, IgG levels were stable from baseline (8.9 ± 2.3 g/L), 3-6 months (10.2 ± 2.2 g/L) and 9-12 months (9.9 ± 2.3 g/L), *P*= 0.11 (mean ± SD, Unianova). When reviewing IgG levels for those patients that switched from IVIg to fSCIg (n=7) separately, we found that six patients had IgG measurements taken after baseline. There were no significant decrease in IgG in these patients from baseline (9.1 ± 3.9 g/l) compared to levels measured at 3-12 months (10.5 ± 2.2 g/l) after initiating fSCIg (*P*= 0.73 [median ± IQR], **Supplementary Table S3** and **Supplementary Methods**). For comparison, in patients switching from IVIg to conventional SCIg (n=28) IgG levels were stable from baseline (9.0 ± 3.7 g/L), 3-6 months (9.2 ± 2.9 g/L) and 9-12 months (8.6 ± 2.3 x g/L), *P*= 0.85 (mean ± SD, Unianova).

### AEs of fSCIg Treatment

The nurses registered side effects and AEs at every visit. After the training sessions for HyQvia were finished, the patients had a follow-up appointment at the Medical Day-unit after 3-4 months and then every 6-12 months depending on the severity of their immunodeficiency. Patients were followed-up for a median of 18 months (IQR 12, range 0–40) after starting fSCIg therapy. The most common side effects reported were: “rubor around injection site” (n=48, 89%), “pressure on injection site” (n=16, 30%) and “itching” (n=12, 22%). In addition, the patients reported a “local burning sensation” (n=7, 13%), pain (n=6, 11%), “injected fluid sinking down to genitalia/groin” (n=5, 9%), tenderness (n=4, 7%), bruising (n=3, 6%), tiredness the day after infusion (n=3, 6%) and feeling unwell (n=2, 4%). No patients experienced severe infections during the switching process, and no patients had severe AEs (grade 3-4).

### Reasons for Termination of fSCIg and SCIg Therapy

Thirteen out of 54 patients (24%) discontinued fSCIg therapy and one was lost to follow-up during the observation period (01.01.2017- 26.05.2020). The duration on fSCIg for the 13 patients who discontinued therapy (calculated from the first training session) was a median of 109 days (IQR 325), range 1-1013 days. There was only one patient that stopped fSCIg before the training sessions were finished. After discontinuing fSCIg, eleven patients switched to conventional SCIg, one switched to IVIg and one stopped Ig therapy altogether.

Also, twenty-one (25%) of the patients that received conventional SCIg training in 2017-2019, discontinued therapy during the observation period (01.01.2017- 26.05.2020). Eleven patients switched to HyQvia and eight patients to IVIg. One patient decided to quit Ig therapy all together, and one was lost to follow-up. The reasons for discontinuing fSCIg therapy are given in **Table 2**. Compared to conventional SCIg, significantly more patients on fSCIg stopped Ig therapy due to local AE (*P*= 0.033) and cognitively/psychological complications (*P*= 0.003, **Table 2**).

**Table 2:** Reasons for discontinuation of fSCIg and SCIg from 2017-2019.

Reasons for discontinuation of therapy	fSCIg, number of patients (n=13)^b^	SCIg, number of patients (n=21)^c^	P-value^d^
**Local AEs (total)** ** ** *Swelling of genitalia/groin^a^* * Local pain/discomfort around injection site* * Bruising* * Itching* Rash	**9** *3* *3* *1* *1* *1*	**4** *0* *1* *1* *1* *1*	0.033
**Systemic AEs (total)** * Flu-like symptoms (fever/body pain)* * Fall in blood pressure* * Fatigue* * Muscle pain* * Tachycardia and dyspnoea* * Nausea*	**3** *2* *1* *0* *0* *0* *0*	**5** *0* *0* *2* *1* *1* *1*	1.000
**Cognitively/Psychological complications (total)** * Found the procedure difficult/unable to learn* * Feeling insecure to self- administrate fSCIg* * Psychological difficult with a new pump*	**6** *4* *1* *1*	**0** *0* *0* *0*	0.003
**Failure of therapy (total)** * Reduced effects on RTI* * Low IgG*	**1** *1* *1*	**6** *4* *2*	0.246
**Other:** *Switched to try HyQvia* *Too little subcutaneous tissue*	**0** *NA* *0*	**12** *11* *1*	0.003

^a^Secondary to injection fluid descending by gravity to the genitals/groin, ^b^Seven patients gave only one reason for determining fSCIg, whereas six patients gave two reasons for determining fSCIg ^c^ One patient gave three reasons to stop SCIg, three patients gave two reasons for discontinuing SCIg.^d^ Fisher’s exact test.

RTI, Respiratory tract infections; AEs, adverse events. The main categories for discontinuation of therapy are given in bold.

## Discussion

This survey describes, to the best of our knowledge, the largest real-word experience with fSCIg, outside of a clinical trial setting. It presents a standardized three-step ramp-up schedule for starting fSCIg with a total follow-up period of 80 patient years. The key findings in this survey are: (i) The main reason for starting fSCIg was that the patients preferred longer intervals for IgRT; (ii) We experienced an increase in training requirement for fSCIg compared to SCIg; (iii) Seventy-four percent of the patients starting with fSCIg wished to continue on this therapy; (iv) The switch from IVIg or conventional SCIg to fSCIg did not reduce IgG levels; (v) The reasons for stopping fSCIg were most commonly local and mild AE, followed by cognitively/psychological complications, whereas systemic AEs were rare.

There are only three previous publications on real-word experience with fSCIg outside a clinical trial setting (7, 13, 14), but only Ponsford et al. have previously compared fSCIg training, in a relatively low number of patients (n=14), with conventional SCIg therapy (13). They found no increase in training-time with fSCIg compared to SCIg, however the training-time for conventional SCIg was higher in the study of Ponsford et al., contributing to the lack of statistical differences. In our study, including a larger number of patients on fSCIg training (n=54), the reason for the increased training-time for fSCIg, even in the treatment-experienced patients, is that the fSCIg-procedure deviated considerably from the SCIg-procedure. Particularly, the increased volume, the injection of hyaluronidase, and the infusion pump were different for fSCIg compared to conventional SCIg. Since we use a shorter training period for conventional SCIg, the increase in training time for fSCIg will have implications for costs and resources used for the training at our department.

Seventy-four percent of patients remained on fSCIg at the end of the observation period, indicating that most patients were satisfied with fSCIg therapy. This was also found in the other studies on real-word experience with fSCIg where 86% (14 patients, median observation time: 6 months) (13), 87% (39 patients, median observation time: 18 months) (14) and 97% (30 patients, median observation time: 39 months) (7) continued on fSCIg therapy at the end of the observation period. The higher satisfaction rates in the above studies, compare to ours, may be due to differences in the groups offered fSCIg therapy. Our survey contained, by far, more patients that were treatment-experienced with conventional SCIg therapy (76%), compared to 35% (14) and 50% (7) in two of the three real-world studies. Ponsford et al. included 71% patients that were treatment-experienced, but studied only 14 patients. The fact that the majority of patients in our study now had experience with both forms of s.c. therapies, and therefore could make a decision based on dual experiences, may have influenced the decision to swop back to conventional SCIg therapy.

For the patients experiencing intolerable local AE after fSCIg therapy, certain measures could be tried before stopping therapy all together e.g. swopping from one to two injection sites, altering infusion rates and/or number of infusion stages. In addition, local measurements such as applying cooling elements or anti-histamine cream could be tried, and if no effect oral antihistamine tablets could be tried before each therapy.

The fall out rate of conventional SCIg was similar as for fSCIg, 25% vs 24%, respectively. However, the patients’ reasons for determining fSCIg were different from conventional SCIg, in that a significantly more patients on fSCIg stopped therapy due to local AE and cognitively/psychological complications compared to conventional SCIg. This is new knowledge that should be taken into consideration when suggesting IgRT for patients.

Wasserman et al. recently reported data from seven US Immunological clinics (11) that most patients received HyQvia differing from prescribing guidelines. One of the most notable variations was the reduced ramp-up periods, which often was reduced to three steps. They concluded that a shorter ramp-up-period did not appear to increase the number of AEs compared to standard ramp-up schedules (11). Accordingly, in our survey there were only three AEs leading to determination of fSCIg that could be considered systemic, and none of them were considered severe. None of the other real-world studies have evaluated a standardized three-step ramp-up period (7, 13, 14).

Unlike SCIg, fSCIg reaches a peak in IgG levels 4-6 days after administration and has a wear-off effect similar to that seen with IVIg (6, 13). It is therefore essential to give a sufficient dose and have an appropriate interval between therapies to avoid that IgG levels become too low before the next dose, potentially making patients more prone to infections. The finding in this survey, of stable IgG levels after switching to fSCIg, supports that it is safe to switch to fSCIg from other Ig therapies.

## Conclusion

A three-step ramp-up dosing schedule for home-based self-administrated fSCIg was safe and well tolerated in the majority of patients but requires longer training time compared to conventional SCIg.

## Data Availability Statement

The original contributions presented in the study are included in the article/**Supplementary Material**. Further inquiries can be directed to the corresponding authors.

## Ethics Statement

Ethical review and approval was not required for the study on human participants in accordance with the local legislation and institutional requirements. Written informed consent for participation was not required for this study in accordance with the national legislation and the institutional requirements.

## Author Contributions

NH, HD, IH, MF, IN, MT, BF, PA and SF collected the data. NH, HD and SJ analyzed the data. The first draft of the manuscript was written by SJ and all authors commented on previous versions of the manuscript. All authors contributed to the article and approved the submitted version.

## Funding

The publication of the article was made possible through funding from the Norwegian Immunodeficiency Society.

## Conflict of Interest

The authors declare that the research was conducted in the absence of any commercial or financial relationships that could be construed as a potential conflict of interest.

## Abbreviations

AEs, adverse events; fSCIg, facilitated subcutaneous immunoglobulin; Ig, immunoglobulin; IgRT, immunoglobulin replacement therapy; IVIg, intravenous Immunoglobulin; IQR, interquartile range; PID, primary immunodeficiency; S.c., subcutaneous; SCIg, subcutaneous immunoglobulin
