# Supplementary material for: Real-World Experiences With Facilitated Subcutaneous Immunoglobulin Substitution in Patients With Hypogammaglobulinemia, Using a Three-Step Ramp-Up Schedule
Source: Front Immunol. 2021 Apr 27;12:670547. doi: 10.3389/fimmu.2021.670547 (PMC8127781; doi:10.3389/fimmu.2021.670547)
Supplement: Supplementary file 1 [file DataSheet_1.docx]

**Supplementary information**

**Real-world experiences with facilitated subcutaneous immunoglobulin substitution in patients with hypogammaglobulinemia, using a three-step ramp-up schedule**

Nina B. Hustad MSc^1^*, Hanna M. Degerud MSc ^1^*, Ingrid Hjelmerud MSc^1^, Mai S.A. Fraz MD^2^, Ingvild Nordøy MD, PhD^2,3^, Marius Trøseid MD, PhD^2-4^, Børre Fevang MD, PhD^2,3^, Pål Aukrust^2-4^, Silje F. Jørgensen MD, PhD^2-4^

*Contributed equally

**Supplementary method**

*Teaching self-administration of subcutaneous immunoglobulin at home*

Self-administration of Ig at home requires sessions teaching patients s.c. infusion technics. This includes managing medical equipment for administration, such as pumps and syringes, and lessons on how to self-inject. In addition, patients need to monitor volume and rate of infusion. At our hospital, we have a standardized training program, mainly run by specialist nurses. It includes information regarding s.c. therapy, practical teaching sessions and support. The aim is to educate the patients so that they can perform the procedure correctly, give them confidence to self-administrate at home and support them throughout the IgRT.

*The adapted version of The American HyQvia dosing guidelines - The three-step ramp-up period*

In cooperation with the manufactures (previously Baxalta, now Takeda) in Norway, we developed an adapted version of the American HyQvia dosing guidelines: The three-step ramp-up schedule. The three-step ramp-up uses 25 %, 50 %, a 100 %, of the total HyQvia dose, given at three different time points. The first dose and the second dose can be given on two consecutive days (or with longer intervals). The third dose is given after the patients have received their electrical infusion pumps from the local ‘Medical equipment supplier’, which often takes a few weeks. The infusion pump needs to be set (e.g. infusion rate) by healthcare professionals and then locked for security before the patients can start self-administrate at home. The major differences between the original guidelines and the three-step ramp-up schedule are that the ramp-up is not given at set weeks (1-7), making the organization of the training more practical, and that the 75% of total HyQvia dose between the dose of 50% and 100% is skipped.

*Infusion time for fSCIg and SCIg*

The three-step ramp-up period is designed to test tolerability, to adopt patients gradually to the large volume injected, and to repeat the training for the patients at least three times. The infusion rates for administration of HyQvia is given below (Table S1a-b). The first two teaching sessions (dose 1 and 2) consists of five steps making the infusion time longer for the two first sessions (Table S1a). In the third teaching session, and if further teaching sessions if needed, 100 % of total dose of HyQvia is given. The recommended infusion rate is given in Table S1b, involving either two or three steps. However, the nurses make individual adjustments to the infusion rates and can increase number of steps in cooperation with the patient. The number of steps and the infusion rate with HyQvia are always tailored to what the individual patient tolerates and is adjusts accordingly.

*Estimating a sufficient and practical dose of fSCIg*

When switching from conventional SCIg or IVIG, to fSCIg the intention was to do no dosing adjustment. We simply calculated the weekly dose of Immunoglobulins and multiplied it with 4 (4 weeks). However, the exact doses of monthly Ig switching from e.g. Hizentra (0,200 g/ml) or Gammanorm (165 ml/ml) to fSCIg are not practical due to the amount of IgG in the HyQvia bottles. Twenty ml Hizentra per week equals 16 g a month, and 40 ml Hizentra every weeks equals 32 g fSCIg. Most patients on 16 g per month was rounded up to 20 g HyQvia (200 ml), and most patients using 32 g Hizentra a month was rounded down to 30 g (300ml) HyQvia a month (Supplementary Table S2). In the beginning of the survey (2017), we used a larger range of doses of fSCIg. However, as we gained more experience with the therapy and based on the feedback from patients starting fSCIg, the preferred dose for most patients became 300 ml (30g) given at 3 weeks (equals 40 g per 4 weeks) or 4 weeks interval depending on previous Ig requirements. The reason for this was practical as the manufacture have a single 30 g package (300 ml), whereas 25 g (200 ml +50 ml), 35 g (300 ml+ 50 ml) or 40g (200ml +200 ml) involve two packages of HyQvia and therefore two bottles of Ig. As a result, there is a need for switching Ig bottles during the procedure. To do this the patients need to time the procedure to avoid air getting into the tube, when the bottle is empty, and ultimately avoid air-bubbles getting into the s.c tissue. If this happen the pump-alarm will go off and the patients need to know the procedure to remove the air-bubbles. In addition, two different hyaluronidase doses from two different vails must be drawn into the syringe to get the full dose required. Overall, more caution must be made when given two separate bottles, and therefore one bottle is preferred by many patients and nurses teaching the procedure. If the requirement is more than 40 g, two packages of HyQvia are prescribed and the procedure to switch bottles is taught by the nurses.

*IgG levels for patients switching from IVIg to fSCIg*

When reviewing IgG levels for patients switching from IVIg to fSCIg (n=7), we included IgG follow up level from 3-12 months after baseline. For those individuals who had two separate measurements between 3-12 months after training, the average value was included (3 out of 6 patients), Supplementary Table S3.

**Supplementary Tables**

**Supplementary Table S1a: Recommended fSCIg infusion rates** **for the first and second training session**

| **Infusion rates for the first^a^ and the second^b^ infusion, maximum speed is 240 ml/hour** | | | | | |
| --- | --- | --- | --- | --- | --- |
| **Step** | **Rate per site ml/ hour** | **Volume HyQvia** | | | |
| **1** | 12 | 2 ml | | | |
| **2** | 30 | 5 ml | | | |
| **3** | 60 | 10 ml | | | |
| **4** | 120 | 20 ml (total 37 ml) | | | |
| **5** | 240 | Remaining volume: | | | |
|  | | *5 g/50 ml* | *10 g/100 ml* | *15 g/150 ml* | *20 g/200 ml* |
|  |  | *13 ml* | *63 ml* | *113 ml* | *163 ml* |

^a^ 25 % of the total fSCIg dose, ^b^ 50% of the total fSCIg dose.

**Supplementary Table S1b: Recommended fSCIg infusion rates for the third training session and future infusions**

| **Infusion rates for the third^a^ and any additional infusions, maximum speed is 300 ml/hour** | | | | | |
| --- | --- | --- | --- | --- | --- |
| **Step** | **Rate per site ml/ hour** | **Volume HyQvia** | | | |
| **1** | 30 | 5 ml | | | |
| *2** | *240* | *40 ml* | | | |
| **3** | 300 | Remaining volume | | | |
|  | | *20 g/200 ml* | *25 g/250 ml* | *30 g/300 ml* | *40 g/400 ml* |
|  |  | *195 ml* | *245 ml* | *295 ml* | *395 ml* |

^a^100% of total fSCIg dose, ^b^ Optional step level

**Supplementary Table S2: Suggested HyQvia dose based on previous Hizentra dose**

| **Hizentra dose (0,2 g/ml) per week** | **Monthly requirement of Ig calculated from Hizentra** | **Suggested HyQvia dose** |
| --- | --- | --- |
| 20 ml | 16 g | 20 g (200 ml) every four weeks |
| 40 ml | 32 g | 30 g (300 ml) every four weeks |
| 50 ml | 40 g | 30 g (300 ml) every three weeks^a^  or 40 g every four weeks |
| 100 ml | 64 g | 60 g (600 ml) every four weeks^b^ |

^a^ Practical dose without the need for switching bottles. ^b^ 300 ml (30g) at two different injection site.

**Supplementary Table S3. Individual IgG values after switching from IVIg to fSCIg**

| **Patient ID^a^** | **Baseline IgG** | ***IgG after 3-6 months*** | ***IgG after 7-8 months*** | ***IgG after 9-12 months*** | **IgG *after 3-12 months*^b^** |
| --- | --- | --- | --- | --- | --- |
| 1 | 9.2 | *9.7* | *-* | *11.7* | 10.7 |
| 2 | 8.3 | *11.8* | *-* | *-* | 11.8 |
| 3 | 8.1 | *-* | *7.5* | *-* | 7.5 |
| 4 | 9. 0 | *10.8* | *-* | *9.6* | 10.2 |
| 5 | 13.3 | *12.0* | *-* | *10.6* | 11.3 |
| 6 | 11.8 | *9.8* | *-* | *-* | 9.8 |
| 7 | 6.9 | *-* | *-* | *-* | - |

^a^ The ID is randomly allocated. ^b^ For those individuals with to separate measurements the average value was included in the analysis.

**Supplementary figure:**

**Supplementary Figure S1: The doses of Immunoglobulin replacement therapy after finishing training for fSCIg and SCIG therapy**

*Median and Interquartile range. ***<0.0001*

**References**

1. HYQVIA [prescribing information]. Westlake Village. CA: Baxalta US Inc. https://wwwshirecontentcom/PI/PDFs/HYQVIA_USA_ENGpdf. 2020.
